# Design, development and testing of a digitally instrumented slurry pump test rig enabling sustainability‑conscious pump assessment in mining

**DOI:** 10.1016/j.ohx.2026.e00801

**Published:** 2026-05-28

**Authors:** Alan Varghese, Paul Huggett, Evert Lessing, Ghulam Mubashar Hassan, Ali Karrech

**Affiliations:** a University of Western Australia, 35 Stirling Hwy, Crawley WA 6009, Australia; b Metso Australia Pty Ltd, Level 2, 1110 Hay Street, West Perth 6005, Australia; c Metso Corporation, Rauhalanpuisto 9, 02230 Espoo, Finland; d IME Consultants Pty Ltd, Suite 41, Level 4/4 Ventnor Ave, West Perth WA 6005, Australia

**Keywords:** Slurry pumps, Digitalization, Test rig, Sustainability, Mining

## Abstract

Slurry pumps are becoming paramount for the mining and mineral processing industries that heavily rely on them to transport solid–liquid mixtures. Basic test rigs and clear water (as opposed to digitized/smart rigs and slurry) are typically used to validate the functioning of slurry pumps and generate performance curves that can be empirically adjusted for slurry conditions. However, these adjustments are rarely aligned with the actual operating conditions, which may lead to discrepancies between expected and real-world performance. This paper presents the design and development of a slurry pump test rig that is proposed to advance digitalization in the mining sector. Unlike conventional setups, this rig is meant to handle real slurry mixtures. It is equipped with a set of sensors that monitor pressure, temperature, slurry density and flow rate and capture performance continuously. The collected data will be instrumental in developing a digital monitoring of the pump condition and possibly assessing its reliability in real time. By enabling digital performance validation and condition monitoring under controlled yet realistic slurry operating conditions, the proposed test rig provides a practical experimental platform to support energy and water aware pump operation and improved maintenance decision‑making. The digitalization system, consisting of data acquisitions systematic analysis and interpretation, can enhance maintenance strategies, operational reliability, and the overall efficiency of slurry transport in mining operations.

## Graphical abstract

**Figure:**
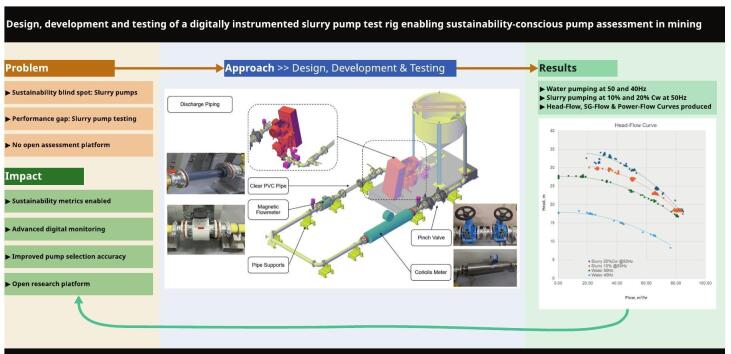

Specifications table.

**Table:** 

Hardware name	Slurry Pump Test Rig
Subject area	• Engineering and materials science • Educational tools
Hardware type	• Measuring physical properties and in-lab sensors • Field measurements and sensors • Electrical engineering and computer science • Mechanical engineering and materials science
Closest commercial analog	No commercial analog is available
Open source license	*CC by 4.0*
Cost of hardware	*AUD 139,685*
Source file repository	*https://doi.org/10.17605/OSF.IO/A87XQ*

## 1 Hardware in context

Slurry pumps are critical for the transport of solid–liquid mixtures in various operations of the mining and mineral processing industry [1], [2], [3], [4], [5]. The hydraulic design of these pumps is inherently complex, as it requires the application of empirical knowledge, engineering expertise, and advanced scientific principles. While traditional design approaches, such as Stepanoff’s 1948 methodology [6], remain foundational, validating the pump performance necessitates rigorous testing under controlled conditions.

Standard testing practices, guided by ISO 9906 [7] and ANSI/HI 14.6 [8], tend to involve clear water to generate baseline performance curves. These curves are later adjusted for slurry applications, though such adjustments often lack validation under real-world conditions, which leads to performance discrepancies [9], [10], [11]. Unlike pump testing, there are no standardized protocols for characterizing slurry transport behaviour, and instrumentation requirements vary based on test objectives, accuracy needs, and test location [12], [13]. In practice, the hydraulic performance of centrifugal slurry pumps is therefore commonly established using water–based factory performance tests, which form the basis of published pump curves. The pump performance under slurry operation is then typically estimated using appropriate correction approaches. While these methods are widely adopted, they do not fully capture the effects of real slurry operation or provide continuous, data–driven insight into pump performance and health under operating conditions [14].

Key measurements include pressure, flow rate, slurry and solids density, temperature, and particle size distribution. Laboratory environments offer precise control and instrumentation, making them preferable to field testing for evaluating hydraulic and mechanical performance, including internal clearances, shaft alignment, seal integrity, vibration, and noise levels. However, many instruments are susceptible to damage or inaccuracy when exposed to abrasive slurry flows [1], [15].

In response to these challenges, both commercial and academic experimental facilities have been developed for slurry‑related testing. Commercial hydraulic laboratories operated by pump manufacturers are typically designed for water pump performance evaluation, materials characterization, and equipment optimization, often under tightly controlled and application specific conditions [16], [17], [18]. In parallel, a substantial body of academic literature exists on slurry pump testing and wear mechanisms, including review studies and experimental investigations employing controlled test rigs, pilot loops, and specialized wear testers to study erosion, abrasion, and erosion–corrosion processes, as well as their dependence on slurry properties, particle characteristics, and operating conditions. While these facilities and studies provide valuable insight into wear processes, performance degradation, and hydraulic behavior, they are generally focused on component level wear characterization, empirical performance correlations, or application specific hydraulic analysis rather than integrated, digitally instrumented condition assessment [19], [20], [21].

Accordingly, experimental facilities must carefully balance measurement fidelity with robustness. While there are comparable experimental rigs designed to test water pumps, slurry mixtures, and pipeline systems, the authors are not aware of existing facilities that combine an open, pump‑centric architecture with continuous, multiparameter digital monitoring specifically for slurry pump operation. The present work therefore proposes a new test rig that provides a digitally instrumented experimental platform designed to enable (i) continuous multiparameter data acquisition under real slurry operating conditions and (ii) the development and evaluation of digital performance and condition‑assessment methodologies that are transferable across pump sizes and installations. This methodological emphasis distinguishes the present work from manufacturer laboratories, which are primarily oriented toward product validation and qualification, as well as from much of the prior academic literature focusing on isolated wear mechanisms, empirical correlations, or application‑specific hydraulic characterization.

The present work does not aim to investigate slurry rheology, flow‑regime classification, pipeline design, or slurry transport scaling. Instead, the focus is on pump‑level performance and condition assessment under slurry operation using digitally enabled measurement and analysis tools.

This paper introduces a custom-designed slurry pump test rig developed to support the digital transformation in mining. The rig is engineered to handle actual slurry mixtures; it integrates a robust sensor array for the real-time monitoring of pressure, temperature, density, and flow. The resulting data are meant to underpin a digital monitoring system capable of assessing pump health and performance dynamically. By combining controlled slurry operation with digitally enabled monitoring, the rig provides a practical experimental platform for evaluating energy‑ and water‑aware pump operation as well as condition‑based performance assessment. This initiative aims to enhance reliability, optimize maintenance, and boost the efficiency of slurry transport systems, which represent a significant progress towards intelligent, adaptive pumping solutions for modern mineral processing operations.

## 2 Hardware description

The presented pump test rig was designed and assembled to analyse the performance of a slurry pump, selected for its common use by the mining industry and its small size compared to other alternatives. The following sections provide a brief overview of the major components.

### 2.1 Test loop

**Fig. 1:**
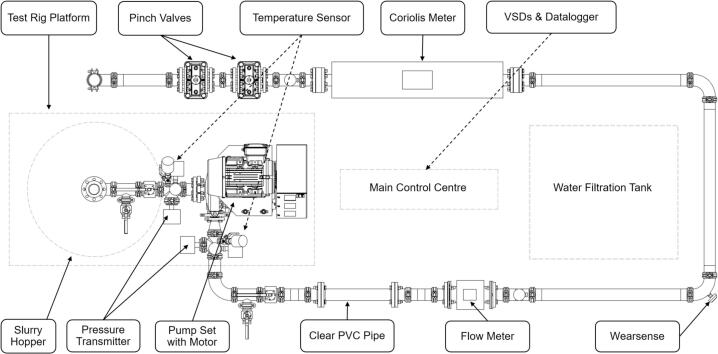
Top view of the test loop.

The test loop shown in Fig. 1 circulates water or slurry through the pump and its accessories, which allows the user to monitor key operational variables such as pressure, flow rate, temperature, density, and vibration. The variables are instrumental in predicting pump performance in real time. Flow conditions within the loop can be adjusted either by modulating the motor speed using the Variable Speed Drive (VSD) or by manipulating the pinch valves installed on the discharge line, thereby artificially increasing or decreasing the system pressure head as required. Slurry composition can be tailored to specific test objectives by modifying particle size distribution, solids density, and concentration.

While the loop is mainly intended for slurry pump performance analysis, utilizing clear water as the working fluid is still possible, and water serves as the carrier medium for slurry. For each test condition, the flow rate is initially set with fully open pinch valves and then progressively reduced to the lowest feasible value. In clear water tests, the flow rate can theoretically reach 0 m^3^/h, but in slurry tests, the minimum flow is constrained by the settling velocity to prevent solids deposition within the loop [4], [22], [23], [24], [25].

As can be seen in Fig. 1, a robust array of sensors is positioned at strategic measurement points to capture and store high-resolution data, including pressure, flow rate, temperature, and density. Based on these measurements, the pump performance can be evaluated accurately, especially in terms of manufacturer’s flow-head (Q-H) curve and efficiency metrics under varying hydraulic conditions.

### 2.2 Test rig

**Fig. 2:**
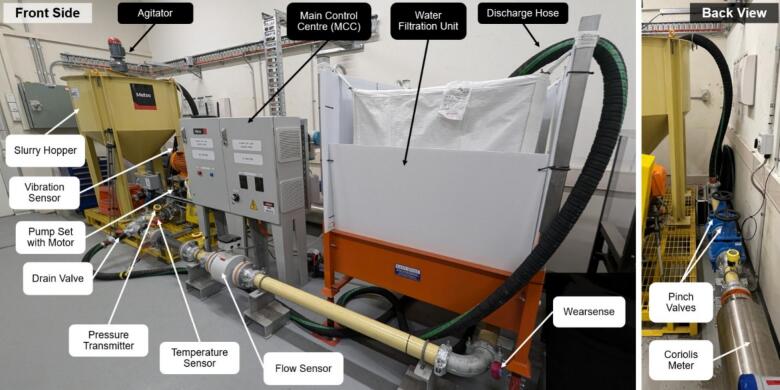
Test Rig with main components.

The main component of the test rig set-up is a Metso HM75 centrifugal slurry pump that is powered by a 15-kW electric motor and connected to a mechanically agitated 700-liter tank, as shown in Fig. 2. The slurry circulates through a DN80 SCH40 carbon steel pipeline before returning to the tank, thereby forming a closed-loop system. The valves in the circuit are adjusted manually to control the flow and pressure of the pumped mixture during testing.

The instrumentation of the rig includes thermocouples, pressure sensors, a magnetic flow meter, a Coriolis mass flow meter, pipe wear sensors, and vibration sensors. All components are selected for compatibility with abrasive and viscous fluids typical of slurry applications. A section of transparent PVC piping is incorporated in the loop to inspect flow behaviour visually. In addition, a water filtration system is included to separate the components (solid and liquid phases) of the slurry when required. The setup is supported by a dedicated Motor Control Center (MCC) and a data interface unit for the data acquisition, monitoring and communication with the instrumentation suite. An emergency stop mechanism is installed to immediately shut down both the pump and the tank agitator in case of a critical event.

### 2.3 Pump assembly

**Fig. 3:**
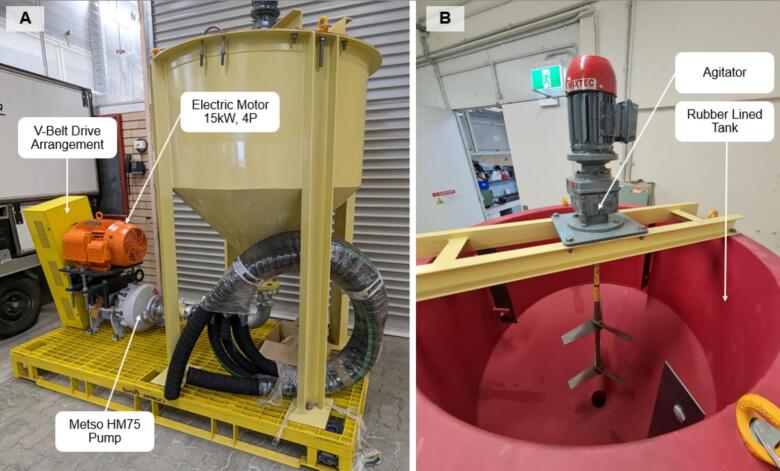
**(A)** Metso HM75 Slurry Pump assembly with 15 kW motor and **(B)** Rubber lined tank with agitator.

The pump assembly shown in Fig. 3A is used to circulate slurry through the test loop. The selected pump is a Metso **HM75 H**orizontal **M**etal centrifugal slurry pump, which has a **75** mm inlet and a 50 mm outlet. It features a single-adjust frame and is equipped with a dynamic expeller seal. The pump utilises a 4-vane closed impeller with a 250 mm diameter and a maximum sphere of 22 mm. Both the impeller and casing are cast from high-chrome alloy, a standard material known for its exceptional abrasion resistance in demanding slurry applications. Mechanical power is supplied by a three-phase motor with a 160 L frame size, rated at 15 kW (50 Hz). The motor is mounted overhead and drives the pump via a V-belt arrangement.

### 2.4 Tank agitator

The 700 L tank functions both as a slurry storage vessel and as the feed reservoir for the loop. To maximize its durability under abrasive conditions, a rubber liner is applied internally in the tank, as shown in Fig. 3B. The tank is equipped with an agitator that is bolted on the top of the support frame; it mixes the solid particles with water effectively, ensures homogeneous suspension of the solid phase within the slurry, and prevents sedimentation buildup within the tank. A VSD is used to adjust the agitator’s rotational speed manually and allow for precise control based on the requirements of the mixing process.

### 2.5 Pressure and temperature sensors

The test rig is equipped with a network of pressure and temperature sensors to monitor system conditions. Pipe spool pieces are installed at both the suction and discharge sides of the pump, each fitted with two pressure and two temperature sensors. Additionally, two more pressure sensors are positioned; one upstream of the magnetic flow meter and another downstream of both the flow meter and Coriolis meter. These sensors enable assessment of pump performance under defined operating conditions, such as discharge flow rate and slurry density.

### 2.6 Magnetic flow meter

The magnetic flow meter uses electromagnetic induction to measure the flow rate through the pipe [26]. A voltage is generated when the slurry moves through a magnetic field while acting as a conductive medium. A key advantage of this technology is its non-intrusive nature; there are no moving parts or obstructions in the flow path, which eliminates pressure drop across the meter, prevents mechanical wear, and makes it especially suitable for abrasive slurry applications.

### 2.7 Coriolis meter

The Coriolis meter measures the mass flow rate and fluid density within the pipeline simultaneously. The Coriolis effect underpins the operating principle of this meter: a moving mass in a vibrating reference frame experiences a force perpendicular to its motion and axis of rotation, which results in measurable oscillations [27]. Usually, the meter consists of two or more tubes that are set into angular harmonic vibration, as fluids flow through them. The Coriolis force causes a slight distortion in the tubes as the fluid passes through. This distortion is directly proportional to the mass flow rate. In addition, the system’s vibration frequency is used to determine the fluid’s density.

### 2.8 Vibration sensors

**Fig. 4:**
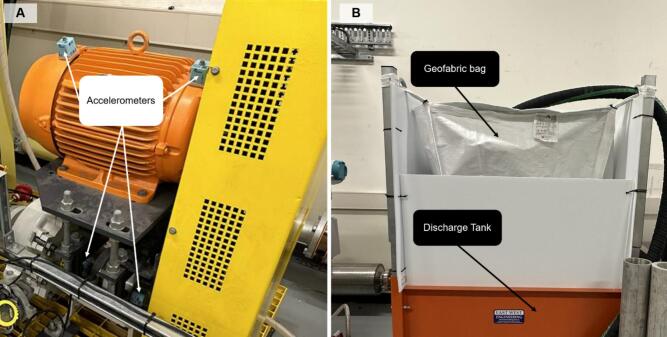
**(A)** Vibration sensors installed on the pump and motor, and **(B)** Water Filtration Unit.

The test rig incorporates wireless accelerometers that monitor the vibration of the pump in order to infer its reliability. These sensors can detect mechanical issues or deviations from the normal functioning of the pump and its motor due to changes in its operating conditions or the integrity of its mechanical parts. Installed at critical points on the pump and motor (Fig. 4), the sensors operate via Bluetooth and transmit data to a dedicated proprietary gateway. The collected vibration data is analyzed through a fit-for-purpose online condition monitoring platform.

### 2.9 Water filtration unit

Following test completion or during maintenance, it is essential to remove slurry from the system to prevent solids from settling in the hopper, piping, or pump. A water filtration unit is used for this purpose. A flexible hose is connected to the drain valve downstream of the pump (Fig. 2), which allows the discharge of the slurry directly into the filtration unit. Solid sand particles are then separated from the slurry using a geofabric bag. The filtered water can subsequently be recirculated into the hopper to assist in further cleaning of the system or reused in future testing.

### 2.10 Motor control center (MCC)

The Motor Control Center (MCC) manages all the electrical components of the test rig. For example, it provides start/stop control for the motors and protects essential devices such as circuit breakers, fuses, overload relays, and an emergency stop to safeguard against electrical faults, short circuits, and overloads. In addition to power control, the MCC includes a variable speed drive (VSD) that regulates the motor speed. Finally, the MCC serves as the central point for system data recording and monitoring.

### 2.11 Control panel

**Fig. 5:**
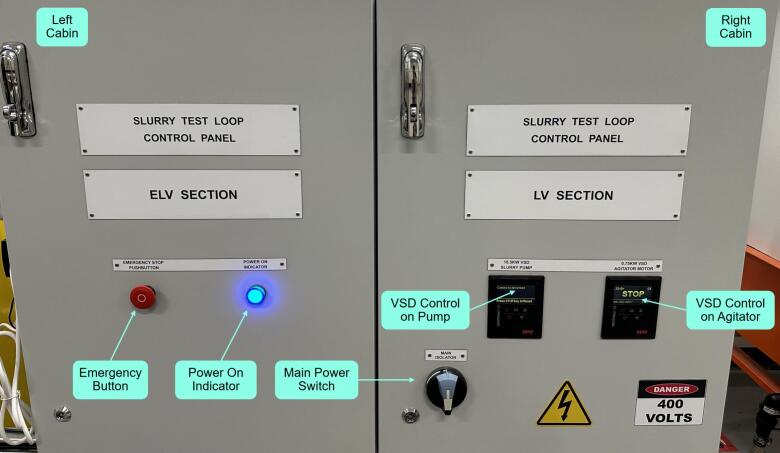
MCC Control Panel with key switches.

The MCC is installed at the ground level with all controls located centrally, which allows operators to quickly and efficiently manage system operations. This layout is practical, and it supports the rapid optimization of the MCC functions. Fig. 5. illustrates the hydraulic control panel and highlights the key switches and their respective functions.

### 2.12 Data interface and communication

The test rig uses a DataTaker DT85 datalogger for data acquisition. The datalogger is located in the left cabin of the MCC panel. It connects via a standard CAT5 RJ45 Ethernet port and collects operational data from the pump and other instruments, which can be accessed and monitored through a PC interface. A data‑flow diagram is provided in the design files that shows sensor signal acquisition, data logging, and analysis within the test rig.

To summarize, the developed slurry pump test rig can support a range of research and engineering (i.e. standard and novel laboratory) tasks, including:

• Investigation of slurry pump hydraulic performance under controlled water and slurry conditions.
• Development and validation of slurry pump condition-assessment and diagnostic techniques using multi-parameter sensor data.
• Evaluation of the effects of slurry properties (e.g., concentration and particle size) on pump behavior and energy consumption.
• Testing and benchmarking of digital monitoring systems and data acquisition methodologies for slurry pumps in mineral processing plants.
• Supporting experimental studies on pump reliability, maintenance strategies, and sustainability-conscious operation.

## 3 Design files summary

**Table:** 

**Design file name**	**File type**	**Open source license**	**Location of the file**
Pump Datasheet	PDF Datasheet	CC by 4.0	https://osf.io/a87xq/files/dbqet
Pump Assembly 3D Model	CAD (stp file)	CC by 4.0	https://osf.io/a87xq/files/k5zhf
Pump Assembly GA	PDF Drawing	CC by 4.0	https://osf.io/a87xq/files/hq9un
Test Loop Assembly 3D Model	CAD (Navisworks)	CC by 4.0	https://osf.io/a87xq/files/a2m78
Test Loop Assembly GA	PDF Drawing	CC by 4.0	https://osf.io/a87xq/files/us58g
Data Flow Diagram	PDF Diagram	CC by 4.0	https://osf.io/a87xq/files/y6e3w
Electrical Diagram	PDF Diagram	CC by 4.0	https://osf.io/a87xq/files/ubxjw
DataTaker DT4DT85 Datasheet	PDF Datasheet	CC by 4.0	https://osf.io/a87xq/files/rgpqk
dEX-2 Set-up 2.05	Application	CC by 4.0	https://osf.io/a87xq/files/8y3af

The Pump Datasheet provides the technical specifications and performance information required to select the correct pump for the system. The Pump Assembly 3D Model provides a detailed, three‑dimensional representation of the entire pump assembly, illustrating its geometry and spatial relationships. Complementing this, the Pump Assembly GA is a general arrangement drawing that outlines the overall layout, key dimensions, and interface points necessary for installation. The Test Loop Assembly 3D Model shows the full configuration of the test loop, including piping, supports, and associated equipment, while the Test Loop Assembly GA provides a general arrangement drawing that defines its layout, footprint, and connection points. The Data Flow Diagram illustrates how sensor signals acquired from the slurry pump and associated instrumentation are routed to the DataTaker DT85 datalogger, transferred via Ethernet communication, and subsequently accessed through a PC interface for monitoring and analysis. An electrical diagram is also provided, detailing the wiring architecture, power distribution, and signal connections between sensors, instrumentation, and the data acquisition hardware. Additionally, the DataTaker DT85 Datasheet summarizes the technical specifications, capabilities, and input/output features of the DataTaker DT85 data logger. Finally, dEX‑2 Set‑up 2.05 refers to the application used to install the dEX software.

## 4 Bill of materials summary

**Table:** 

**Designator**	**Component**	**Number**	**Cost per unit −currency**	**Total cost −** **currency**	**Source of materials**	**Material type**
**Pipe and Fittings** P1	DN80 SCH80 Pipe with Victaulic grooved ends	6	$ 264.87	$1589.23	Con-Mech Materials Handling	Carbon Steel
P2	DN80 clear PVC with CL150 flange both ends	1	$571.18	$571.18	Con-Mech Materials Handling	Clear PVC
P3 with F5 & F6	DN80 SCH40 pipe with victaulic grooved ends. F5 and F6 included in fabrication	2	$174.19	$348.37	Con-Mech Materials Handling	Carbon Steel
P4	DN80 rubber hose	1	$721.83	$721.83	Henderson Hose & Fittings	Rubber
F1	VIC-Flange Adaptor 741 – DN50 to suit CL150 flange	1	$45.58	$45.58	Caddy Industrial	Carbon Steel
F2	Victaulic No.50 – DN80 x DN50 concentric reducer	1	$14.72	$14.72	Caddy Industrial	Carbon Steel
F3	Victaulic No. 60 – DN80 cap (drill & tap ½“ BSPT central)	2	$11.07	$22.13	Caddy Industrial	Carbon Steel
F4	Victaulic No. 20 – DN80 tee with 3x threadolets	2	$518.76	$1037.52	Con-Mech Materials Handling	Carbon Steel
F7	Victaulic No. 100 – DN80 90° long radius elbow	5	$62.15	$310.75	Caddy Industrial	Carbon Steel
F8	Victaulic No 25 – Reducing tee DN80 x DN80 x DN50	2	$50.05	$100.1	Caddy Industrial	Carbon Steel
F9	Victaulic style 77 – DN50 flexible coupling	2	$19.32	$38.63	Caddy Industrial	Carbon Steel
F10	Victaulic No.45R – DN80 ANSI 150 flange adapter – nipple raised face	12	$167.30	$2007.59	Caddy Industrial	Carbon Steel
F11	Victaulic No.48 – DN80 hose nipple	1	$161.13	$161.13	Caddy Industrial	Carbon Steel
F12	Victualic Style 77 – DN80 flexible coupling	30	$26.95	$808.5	Caddy Industrial	Carbon Steel
**Equipment and Valves** E1	Test Loop Tank	1	$13605.90	$13605.90	Acute Fabrication	Rubber Lined / Painted Steel
E2	Metso HM75 Slurry Pump with 15 kW motor Belt Drive	1	$8867.06	$8867.06	Metso	Metal
E3 & E4	RTD with thermowell, ½“ BSP, U = 25 mm with TH300 puck style transmitter, 4-20MA, HAR	4	$1023.44	$4093.76	Instrowest	Metal
E5, E6, E16	Vegabar 82, 0–5 barg, G1/2 process connection, 4-20MA/hart	6	$2,413.68	$14,482.08	Instrowest	Metal
E7	½“ ball valve – BSP thread	6	$18.66	$111.96	Bunnings	Metal
E8	Victaulic Series 726 – DN50 ball valve	2	$427.47	$854.94	Caddy Industrial	Metal
E9	Victaulic Series 726 – DN80 ball valve	2	$932.56	$1865.12	Caddy Industrial	Metal
E10	Siemens DN80 transmag ANSI 150 flange-remote mainspowered	1	$7534.23	$7534.23	Instrowest	Metal
E11 with F13	Hugger wear sensor with M10x1.25x3/4 UNF OD threaded insert C/W adaptor to suit ¾“ UNF threadolet	1	1126.13	1126.13	Metso	Metal
E12 with F14	Optimass 7400 – DN80 coriolis meter (ANSI 150 flange)	1	$33515.90	$33515.90	Krohne	Metal
E13	BE080M10-553-NR DN80 pinch valves (ANSI 150 flange)	2	$3553.00	$7106.00	Ventomat	Metal
E14	Switchboard Complete Control Panel with VSD and Datalogger	1	$23815.00	$23815.00	Dynapump	Metal
E15	8 mm anti-vibration pad	6	$6.16	$36.95	Clark Rubber	Rubber
**Pipe Supports** S1	2 T Pallet	1	$2154.9	$2154.9	Daywalk	Steel
S2, S3 & S4	DN80 pipe support with DN80 pipe clamp packing plates to suit pump bolt profile	8	$535.15	$4281.20	Acute Fabrication	Steel
**Additional Equipment** Water Filtration Unit	Water Filtration Tank and Geo-fabric bag	1	$1958.00	$1958.00	East West Engineering	Steel
Portable Water Pump	Small Pump to recirculate water back to tank	1	$169.00	$169.00	Bunnings	Metal
Filtration Pump Hose	Transfer hose from filtration unit to tank	1	$80.30	$80.30	Bunnings	PVC
Agitator	Mixtec	1	$3547.50	$3547.50	Mixtec	Metal
Vibration Sensor Solution Kit	4 Sensors and 1 Gateway solution	1	2702.70	2702.70	Dynamox	Metal

Note: Prices are in AUD and include GST (10%).

## 5 Build instructions

**Fig. 6:**
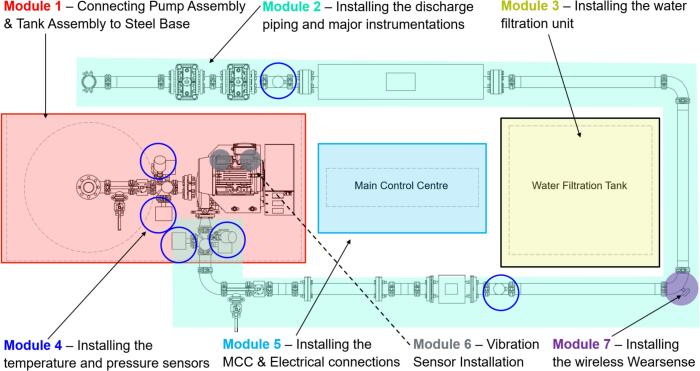
Modular construction breakdown of slurry test rig.

This section outlines the modular assembly of the slurry test loop, as illustrated in Fig. 6. Standard workshop and laboratory tools are sufficient for construction. The listed components and equipment are directly related to the rig’s function and operation. Instructions focus on the modular setup used in this configuration.

### 5.1 Slurry test rig

1) Safety instructions for the facility are verified, and the appropriate Job Safety and Hazard Analysis is completed before any work begins. Once all safety requirements are confirmed, a suitable area is arranged for building the test rig in modular sections.

### 5.2 Module 1 – Connecting pump assembly & tank assembly to steel base

1) Slurry Hopper

The 700 L slurry hopper is secured onto the steel base, providing a stable, level platform that allows easy transport into the laboratory. The hopper is supported by four welded carbon‑steel legs, ensuring structural integrity and proper alignment on the base.

2) Agitator

**Fig. 7:**
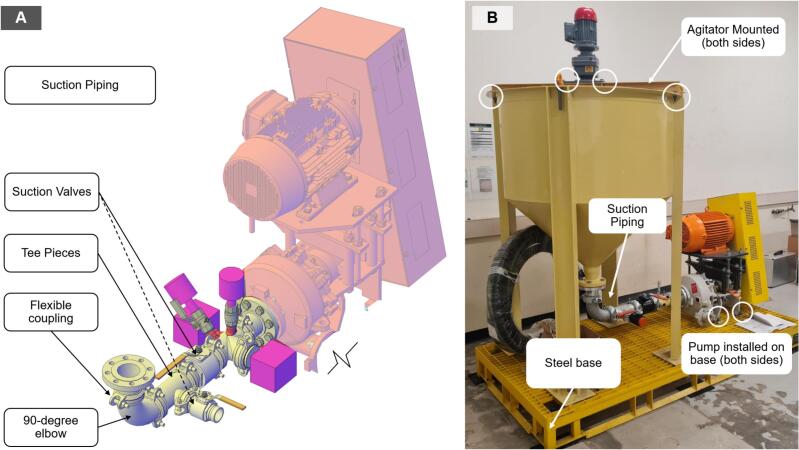
**(A)** Suction piping layout and **(B)** Installed agitator, suction piping and pump.

The agitator frame is bolted to both sides of the hopper opening to provide structural support. The agitator is then mounted in an inverted position on the frame, enabling top‑down mixing of slurry within the hopper (Fig. 3B and Fig. 7B).

3) Suction Piping Connection

Connection of the suction piping begins at the hopper flange. The flexible coupling is attached first, followed by the 90‑degree long‑radius elbow. Installation continues with the reducing tee pieces, ball valves, and additional flexible connections as shown in Fig. 7A. All joints are checked to ensure proper sealing, preventing leaks and maintaining system integrity.

4) Pump Assembly Installation

The pump assembly is mounted onto the steel base and aligned with the suction flange on the piping. Before finalizing installation, the pump’s position and alignment on the base are verified to avoid operational issues. The pump is then secured with bolts tightened to the specified torque, ensuring a fixed position during slurry operations. Correct torque application minimizes vibration and prevents misalignment during operation.

### 5.3 Module 2 – Installing the discharge piping and major instrumentations

1) Pipe Supports

**Fig. 8:**
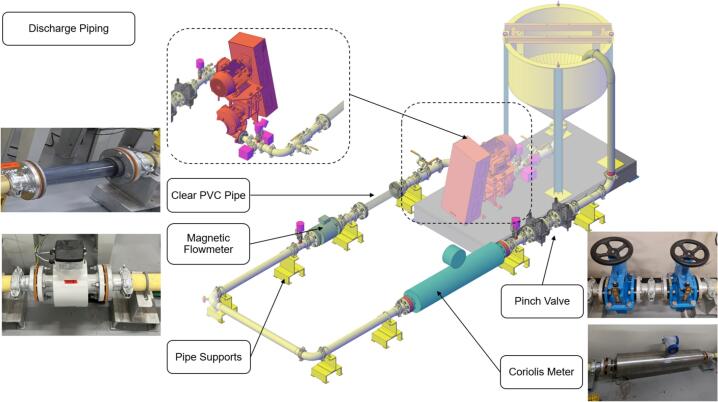
Discharge piping and major instrumentation installation layout.

The installation process begins by placing eight pipe supports on the ground at designated locations where the discharge piping requires additional stability. These supports are necessary in areas where the steel base cannot reach the piping, as shown in Fig. 8. Each support is aligned and stabilized to ensure proper placement as piping installation progresses.

2) Immediate Discharge Piping Components

Piping installation begins at the pump discharge, where the flange is connected to a concentric reducer to achieve smooth flow transition. Victaulic flexible couplings are then used to attach the tee piece, followed by grooved‑end PVC pipes that lead to a carbon‑steel elbow, forming a loop. All flanged connections are sealed to maintain a leak‑free system. For proper alignment and stability, the piping is secured to the supports using bolts, washers, and nuts at the required locations.

3) Clear PVC Pipe

A section of clear PVC pipe is installed in the discharge line upstream of the magnetic flow meter (Fig. 8). This transparent segment allows visual inspection for contamination, air bubbles, inconsistent flow, or slurry settling. It is a valuable tool for monitoring slurry concentration and confirming proper flow conditions.

4) Magnetic Flow Meter

Discharge piping installation continues from the clear PVC section by connecting the remaining pipe components before installing the magnetic flow meter. The flow meter is mounted horizontally to ensure accurate flow measurement, with straight inlet and outlet sections maintained. Avoid installing the flow meter in pipe sections with a free outlet that may run empty, or at the highest point of the piping system, where gas accumulation could interfere with its readings. To ensure accurate measurement, the sensor is installed at the same electrical potential as the medium, providing potential equalization between the medium, pipe, and transmitter.

The transmitter is mounted on the steel‑framed column behind the control panel using the supplied mounting plate and fastening brackets. The transmitter is then connected to the sensor with the shielded sensor cable. The cable shield is bent over the clamping piece of the cable gland, after which the clamping piece is inserted into the threaded bushing with a slight right‑hand turn. Finally, the locknut is tightened until the cable is firmly secured.

5) Remaining Piping installation up to the Coriolis Meter

The discharge piping installation continues as per the General Arrangement (GA) drawing, with each section positioned to match the specified layout. Victaulic couplings, elbows, and straight pipe sections are assembled sequentially to form the required pipeline path. Throughout the process, all pipe supports are correctly positioned and securely fastened to maintain system alignment and stability.

6) Coriolis Meter

The Coriolis meter is installed horizontally on the discharge line using flange connections. Due to the weight of the meter, it is supported and aligned carefully to avoid stressing the piping. Proper installation ensures accurate flow measurement and long‑term structural reliability.

7) Remaining Piping and Pinch Valves installation

The final section of the discharge loop is completed following the GA drawing. The first pinch valve is installed downstream of the Coriolis meter, functioning as a shut‑off device that allows zeroing of flow under full operating pressure and temperature. A second pinch valve is installed afterwards to prevent reverse flow when the pump is shut down.

Installation continues with flexible hoses at the end of the loop to return flow to the hopper. These hoses are secured to the top of the hopper using appropriate fittings. Throughout the process, all piping is properly aligned and firmly supported using pipe supports, bolts, washers, and nuts to ensure overall system integrity.

### 5.4 Module 3 – Installing the water filtration unit

Once testing is complete, it is recommended to remove the slurry from the system, as it can settle inside the hopper, suction, and discharge piping. A water filtration unit, as shown in Fig. 4B is used to facilitate this removal process. The unit filters out sediment (e.g., sand) from the slurry. The filtration unit is a standalone assembly, featuring a suspended filtration bag mounted on a frame, an integrated water holding tank with a 50 mm drain valve and forklift guides for easy relocation

1) Discharge tank

After building the entire test rig, the discharge tank is placed at the end of the system to collect effluent without leakage. During installation and operation, the drain valve of the filtration unit is kept closed to prevent accidental spillage.

2) Flexible Hoses

**Fig. 9:**
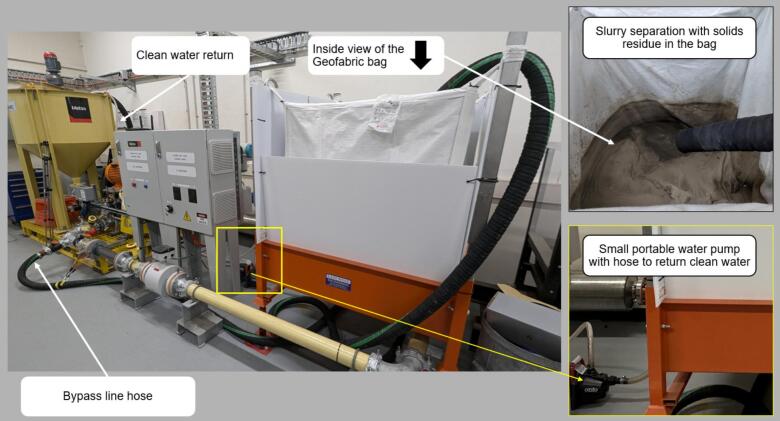
Setup of the water filtration unit showing the bypass line, pump, and geofabric bag.

As shown in Fig. 9, a flexible hose connects the filtration tank outlet to the slurry hopper, and another hose links the outlet of the discharge tank to the inlet of a small portable water pump. All hose fittings are tightened to prevent leaks and maintain a continuous flow of filtered water back into the system during operation.

3) Geofabric Bag

A geofabric bag is placed inside the discharge tank as shown in Fig. 9. This bag filters out sand and solid particles and allows water to drain through and return to the hopper. Corrugated plastic sheets are used to prevent splashing slurry over the surrounding area.

4) Bypass Line Hoses

A flexible hose is connected to the drain valve located on the main discharge line after the pump to create a bypass line for draining slurry after testing. All hose connections are securely tightened to prevent leaks. The other end of the hose is positioned inside the geofabric bag and secured firmly to avoid backflow or splashing during operation.

### 5.5 Module 4 – Installing the temperature and pressure sensors

A pipe spool piece is installed directly at both the suction and discharge ends of the pump. Each spool piece is equipped with two thermocouples and two pressure sensors. In addition, two more pressure sensors are positioned as shown in Fig. 6: one upstream of the magnetic flowmeter and the other downstream of the Coriolis meter. These sensors collectively monitor the key variables of the rig, including the discharge flow rate, slurry density, temperature, and pressure, which are essential for evaluating pump performance under varying operating conditions.

### 5.6 Module 5 – Installing the MCC & electrical connections

1) MCC

The MCC control panel box is placed at the center of the test rig, where it is anchored to the floor, as shown in Fig. 2. The selected location provides unobstructed access to the controls for easy operation and maintenance.

2) Electrical connections

**Fig. 10:**
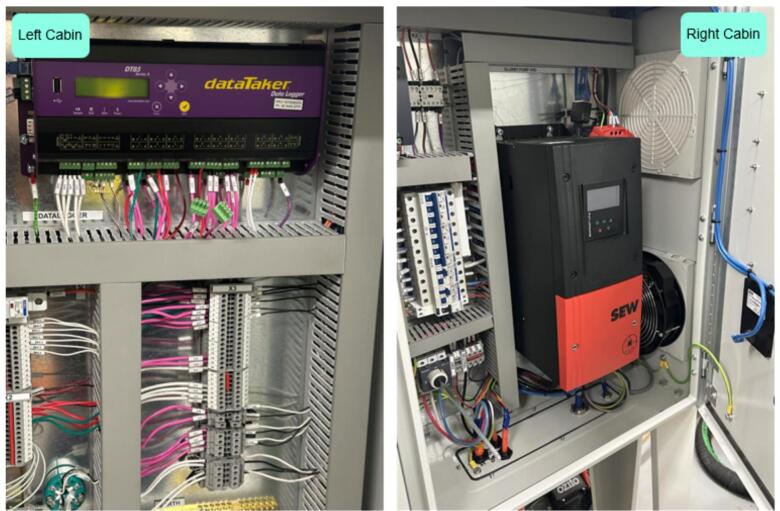
Wiring inside the MCC left and right cabinets.

Internal electrical wiring and connections, including power and signal cables, are routed and labelled in accordance with the electrical schematics. Fig. 10 serves as the reference for the complete wiring inside the MCC, including connections to the Datataker and Variable Speed Drives (VSDs).

The datalogger and Ethernet connection are located on the left-hand side of the MCC panel and are accessed via a standard RJ45 connector. All instrumentation data are recorded either to the Datataker (as the primary data source) or to the proprietary gateway, where applicable.

### 5.7 Module 6 – Vibration sensor installation

Wireless sensors that contain accelerometers and thermocouples are used to monitor the vibration and surface temperature of the pump and its motor. These sensors communicate via Bluetooth with a proprietary gateway that transmits the data to a cloud-based system, which offers a continuous acquisition of electrical signals and reflects its operating conditions.

1) Surface Preparation and Sensor Mounting

The areas of interest are cleaned thoroughly to remove grease and strip any paint for proper adhesion before mounting the sensors. Adhesive is then applied to the base of the stainless steel mounting plate, which is placed firmly onto the designated location. Approximately 5 minutes are allowed for the adhesive to set. Once the base is stable, the sensor is screwed onto the plate. If the sensor cannot support itself immediately, it is held in place for at least five minutes until the adhesive cures fully.

2) Connect Sensors to Gateway and system setup

The gateway is activated by entering its serial number and PIN into the system. Then, each sensor is connected to the gateway via Bluetooth by scanning the QR code on the sensor. After establishing the connection, the sensor’s position is configured within the system for accurate data collection and analysis.

### 5.8 Module 7 – Installing the wireless wearsense

A proprietary Metso wear sensor (WearSense), as shown in Fig. 6, is installed on the test rig to assess whether the technology could be effectively integrated into slurry pipe loops while maintaining leak‑free performance under operational conditions. Although WearSense is traditionally used in dry processing applications, most commonly monitoring wear in chutes, the purpose of this test is to assess its suitability in a slurry environment.

For this assessment, the sensor is fitted by drilling and tapping the elbow section of the piping, after which the device is securely fastened in place. However, the actual wear‑measurement capability of the sensor falls outside the scope of this module. Significant material wear on the pipe loop is not expected during the testing period, and full functionality requires a portable tablet for data collection and analysis, which is not included in the current setup.

## 6 Operation instructions

Pre-operating checks need to be conducted to verify that the system is functional, accurate, and safe to use before starting the rig.

### 6.1 Pre-operating checks

a) Physical components such as piping and connections should be inspected to identify any signs of leaks, damage, or loose fittings. All valves should be positioned correctly (open or closed as required) and operated smoothly. Pumps must be inspected for wear, damage, seals, and lubrication levels to be adequate. Instruments and sensors, including the flow meter, Coriolis meter, pressure sensors, thermocouples, and accelerometers, should be mounted properly and connected securely.
b) Safety equipment must be examined to verify that emergency stop mechanisms are functional, safety guards are in place, and all operators are equipped with the appropriate personal protective equipment (PPE).
c) Electrical and control systems should be verified to ensure that the power supply and electrical systems are properly grounded. Similarly, the Motor Control Center (MCC) should be examined to verify that it is operating correctly. Emergency shutdown procedures must be tested to ensure they activate reliably and bring the system to a safe stop when needed.
d) Flow path configuration should be confirmed to match the design specifications. For example, the system should be inspected for any blockages or restrictions that could compromise the accuracy of the test or cause damage to the rig.
e) Instrumentation and software readiness must be verified by ensuring that all instruments are calibrated correctly and function as intended. For example, the operator should confirm that the laptop is prepared with the Dex system open and ready for operation.

### 6.2 Startup and operation

**Fig. 11:**
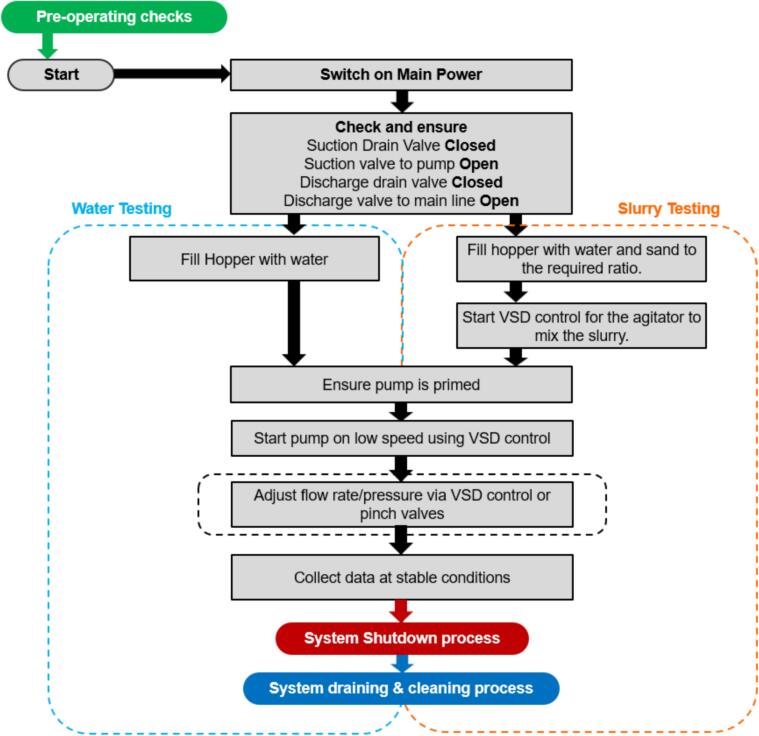
Operating procedure flowchart.

An overview of the key steps in the operation procedure is presented in the following flow chart (Fig. 11).

a) The operation of the system begins by switching on the main power isolator located on the electric control panel. Activating this isolator supplies power to both the agitator and the slurry pump, enabling the system to function. Before water or slurry is added to the hopper, several checks are carried out to ensure the setup is correct. The suction drain valve is closed while the suction valve to the pump in open. On the discharge side, the discharge drain valve remains closed and the discharge valve to the main line is open for pumping.
b) The procedure varies depending on whether the test being carried out involves water or slurry. For water testing, the hopper is filled with clean water to the required level specified for the experiment. In contrast, slurry testing requires preparing a mixture of sand and water in the hopper according to the designated test ratio. The VSD control for the agitator is started to initiate mixing, ensuring the solids remain uniformly suspended before pumping begins. Throughout the filling process, the system is continuously monitored to prevent spills or overfilling.
c) Before the system is started, the pump is checked to ensure it is fully primed with either slurry or water. Proper priming prevents the pump from running dry, which could cause significant damage to the internal components. Once priming is confirmed, the pump is started at a low speed using the VSD control, allowing the water or slurry to circulate gradually through the system. As flow becomes stable, the pump speed is increased in stages until it reaches the required operational level. Throughout this process, the system is closely monitored for any signs of malfunction or distress.
d) Adjust flow rates and pressures using the control system. Ensure the system stabilizes at the desired conditions before proceeding with testing or making further adjustments. Once stable operating conditions are achieved, the required data is collected in accordance with the established test protocols, ensuring that measurements reflect consistent and reliable system behaviour.

### 6.3 Control system

The system features only a limited degree of automatic control, as most operational inputs are intentionally kept manual to support flexibility during testing. For clarity, the control system can be divided into several functional components, each contributing to the overall operation of the test rig.

a) Flow control within the system is primarily managed through the VSD connected to the pump, which enables manual adjustment of the pump’s rotational speed and, in turn, the overall flow rate. A magnetic flow meter (FIT‑01) installed on the loop provides continuous measurement of the volumetric flow of slurry as it circulates through the system. Complementing this, a Coriolis meter (X‑03) supplies both real‑time slurry density data and an additional flow‑rate measurement, offering a secondary verification point for monitoring accuracy. All data generated by these instruments are recorded in the Datataker.
b) Slurry density is monitored using the Coriolis meter (X‑03), which provides continuous and accurate measurement of the mixture’s density as it flows through the system. These density values are automatically logged in the Datataker.
c) System pressure is primarily managed through the use of pinch valves (PI‑001‑11 and PI‑001‑12), which can be adjusted to increase the pressure head within the loop when required. Six pressure indicators, labelled PIT‑01 through PIT‑06, are distributed across the system to track pressure at key locations. Each indicator is set to operate within predetermined pressure ranges, allowing the system to capture variations and monitor changes throughout operation. All pressure readings from these sensors are continuously logged by the Datataker.
d) Temperature within the system is monitored using four temperature indicators (TT‑01, TT‑02, TT‑03, and TT‑04), each positioned at key locations along the loop. These measurements provide insight into temperature changes as the slurry or water circulates through the system. These readings are also logged via the Datataker
e) Electrical power to the system is managed through the motor VSDs that control both the pump and the agitator. These VSD units are fully integrated into the main control system, allowing regulation of electrical parameters such as current, frequency, and overall power consumption during operation. Electrical parameters are monitored and logged through the Datataker.

### 6.4 System shutdown

Before initiating the shutdown sequence, all collected test data is verified to ensure it has been properly logged and saved, and the system is confirmed to be operating under stable conditions. The flow rate is then gradually reduced using the MCC, allowing the system to adjust smoothly and preventing any pressure surges. As the flow decreases, other operational parameters, such as the agitator speed, are carefully lowered to their minimum settings or switched off in a controlled manner. Once the flow rate reaches its lowest operational level, pump circulation is stopped, and the remaining auxiliary equipment is shut down sequentially. To prevent sediment buildup, the system is then purged with water to flush out residual slurry from the discharge piping and associated components, ensuring that the resulting liquid is disposed of or recycled according to environmental and safety regulations. The appropriate drain valves are opened to direct slurry through the bypass line into the water filtration unit, following the draining and cleaning procedures described in Section 6.5. After the system has been fully drained, a final visual inspection is carried out to identify any signs of wear, damage, or leakage that may have occurred during operation. The entire shutdown process, along with any issues encountered, is documented to ensure a complete operational record is maintained.

### 6.5 System draining and cleaning

This procedure is used to drain and clean the slurry test loop using the filtration unit and is carried out only when the slurry requires replacement or when the system will be out of operation for an extended period. The process begins by shutting down the pump system following the instructions in Section 6.4 and ensuring that the bypass hose is positioned securely at the top of the filtration unit. Once the system is safely shut down, the discharge isolation valve is closed, and the agitator is started to ensure the slurry within the tank is properly suspended before transfer.

With the slurry properly mixed, the pump is started at a low flow rate to begin moving the slurry into the filtration unit. As the filtration tank begins to fill, the return pump is activated to send the filtered water back to the system tank. Throughout this stage, care is taken to prevent overflow of the filtration tank and to ensure that the system tank does not run dry. The filtration tank level is monitored closely, and the return pump is stopped once the tank approaches empty. After this, the filtered water is allowed to circulate through the loop for several minutes to flush residual slurry from the system.

While the discharge pump remains running, the drain valve is gradually opened and the isolation valve is slowly closed to direct flow appropriately during flushing. This process may be repeated as necessary to ensure thorough cleaning. Once the sand has been fully removed by the filtration unit, the isolation valve is closed again, and the pump is used to remove the remaining water from the hopper. The filtered water is drained into the filtration unit for disposal, although a small amount of water is intentionally left in the system to prevent dry running. The pump is then shut down according to the standard procedure.

To complete the process, both the tank and pump drain valves are opened to fully empty the system. Any remaining water is collected and either disposed of responsibly or stored for future use, depending on operational requirements.

### 6.6 Calibration and maintenance schedule

To ensure reliable operation and measurement accuracy of the slurry pump test rig, routine calibration checks and maintenance activities are implemented as part of standard operational practice. The calibration and maintenance schedule for key mechanical components and instrumentation is summarized in Table 1, Table 2.

**Table 1:** Routine Maintenance Schedule.

System / Component	Maintenance Activity	Action	Frequency
Emergency Stop System	Functional check	Check / Repair	10 h (Daily)
Paint / Surface Finish	Inspect for damage	Check / Repair	50 h (Weekly)
Piping connections & flanges	Inspect for leaks, gasket wear	Check / Tighten / Repair / Replace	10 h (Daily)
Electrical system	Inspect for defects	Check / Repair	50 h (Weekly)
Loose parts (nuts, bolts)	Mechanical integrity check	Check / Tighten	10 h (Daily)
Valves & instruments	Condition inspection	Check / Repair / Replace	50 h (Weekly)
Pipe supports	Structural inspection	Check / Repair	50 h (Weekly)
Rubber hose	Inspect for wear or damage	Check / Replace	10 h (Daily)
Pump bearings	Noise, vibration, overheating	Check / Repair / Replace	50 h (Weekly)
Pump seals	Inspect for leaks	Check / Replace	50 h (Weekly)
Pump–motor coupling	Alignment check	Check / Repair	50 h (Weekly)
Tank surface / paint	Inspect for damage	Check / Repair	50 h (Weekly)
Tank rubber lining	Inspect for wear	Check / Repair	50 h (Weekly)
Agitator structure	Inspect finish, bolts, supports	Check / Tighten / Repair	50 h (Weekly)
Agitator impeller	Inspect for wear or damage	Check / Repair	50 h (Weekly)

**Table 2:** Instrument Calibration and Maintenance Guidance.

Instrument Type	Calibration / Verification Trigger	Likely Cause of Error	Corrective / Maintenance Action
Temperature sensors (RTD)	Incorrect or unstable readings	Incorrect calibration	Check calibration regularly; recalibrate as required; monitor drift; replace if necessary
Pressure sensors	Deviating pressure readings	Wiring or connection issues	Inspect wiring and connections; repair or replace if required
Magnetic flow meter	Incorrect flow readings	Worn or dirty sensor	Inspect for slurry buildup; clean and replace if required
Coriolis meter	Density or mass flow deviation	Sensor fouling or wear	Inspect sensor; clean buildup; replace if required
All sensors	Signal loss or instability	Power supply issues	Verify power supply is present and stable

## 7 Validation and characterization

### 7.1 Slurry pumping

The test rig is designed to accommodate slurry pumping operations. The rig is capable of handling slurry conditions that allow stable centrifugal slurry pump operation, with the practical operating envelope governed by the pump used rather than limitations of the test loop itself. For the HM75 heavy‑duty slurry pump employed in this study, manufacturer recommendations indicate operation up to approximately 40% solids concentration by volume (Cv), subject to slurry properties and operating conditions. Particle size capability is governed by the maximum sphere size of the impeller (22 mm), and to avoid solids bridging or cramping phenomena, a commonly adopted guideline is to limit maximum particle size to approximately one‑third of this value, corresponding to around 7 mm. After initial water commissioning, sand was gradually introduced in 10 kg increments until the slurry concentration reached approximately 25% by weight. Several pumping cycles were conducted to ensure a smooth transition and achieve stable operation. Once stability was confirmed, the pump speed was adjusted using the Variable Speed Drive (VSD) to evaluate system control. Visual inspection through a transparent pipe allowed observation of slurry movement during speed variations.

**Fig. 12:**
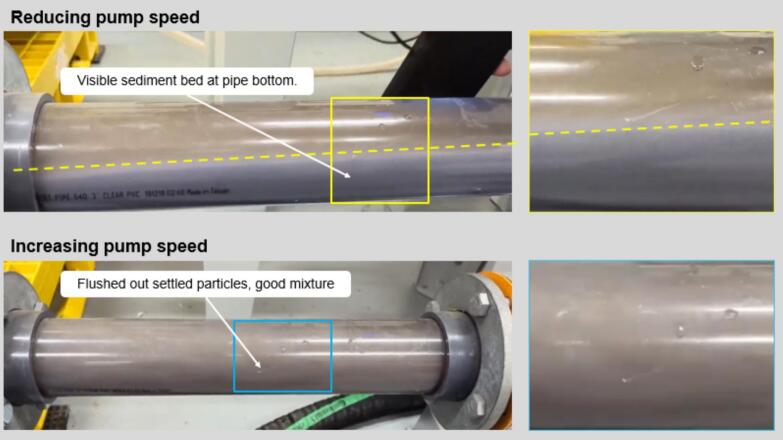
Effect of line velocity on slurry flow control using the test rig.

Reducing the pump speed demonstrated sedimentation tendencies when the line velocity was insufficient. Subsequently, increasing the speed effectively flushed out the settled particles and confirmed the system’s responsiveness to operational changes. Fig. 12 illustrates these stages and highlights the ability to control slurry flow as required. It is important to note that slurry pumping inherently exhibits higher variability due to factors such as concentration changes, particle degradation, and other dynamic effects [9], [14], [28]. Accordingly, the present work does not aim to characterise slurry rheology, solids transport behaviour, flow‑regime transitions, or slurry pipeline scaling effects, as these fall outside the intended scope of the test rig. In this context, understanding behavioural patterns is more critical than achieving absolute measurement precision. A detailed analysis of operational conditions will be presented in a separate publication.

### 7.2 Pump performance

Four tests were conducted to establish a performance benchmark and assess repeatability. The first two tests involved water at different speeds (50 Hz and 40 Hz), which generated the performance curves from zero-flow conditions to fully open positions by gradually adjusting the pinch valves. Flow was increased incrementally to capture the full curve.

Subsequently, slurry tests were performed at 50 Hz with two concentrations: 20% and 10% by weight (Cw). For the 20% Cw test, 100 kg of sand was added in 10 kg increments to the calculated water volume. The slurry was circulated for several minutes to ensure proper mixing. Unlike water tests, slurry tests began with the valve fully open and then progressively reduced flow to approximately 20 m^3^/hr, while collecting data at each step. Zero-flow conditions were avoided to prevent line blockage. After completing the 20% Cw test, the loop was flushed, refilled with water, and a 10% Cw slurry was prepared by adding 45 kg of sand in similar increments. Data were collected following the same procedure.

**Fig. 13:**
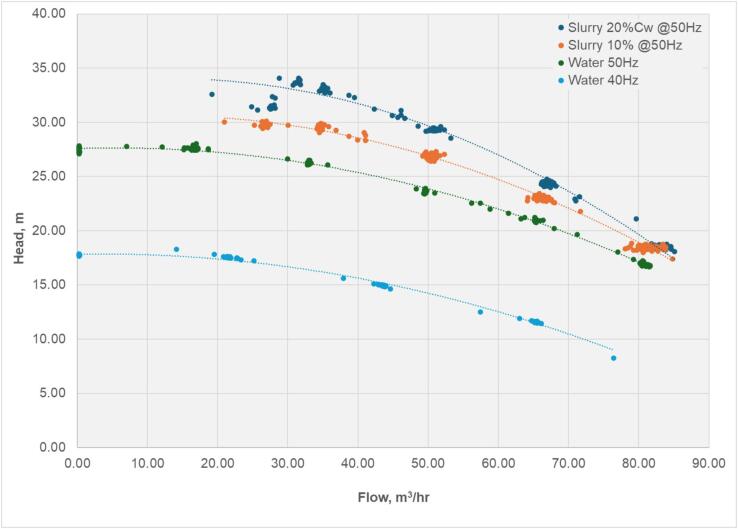
Head vs Flow Curve for Slurry and Water Tests.

**Fig. 14:**
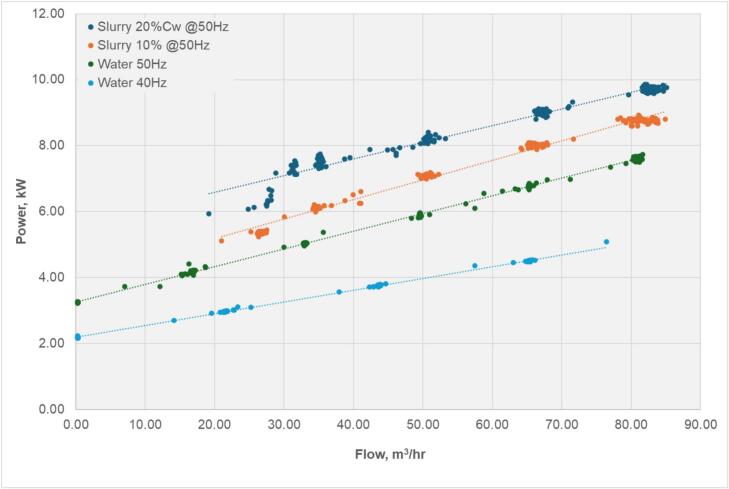
Power vs Flow Curve for Slurry and Water Tests.

**Fig. 15:**
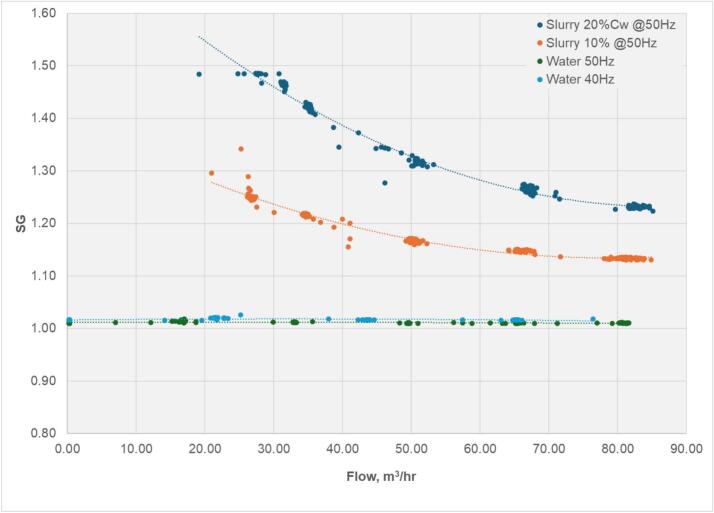
SG vs Flow Curve for Slurry and Water Tests.

Fig. 13, Fig. 14, Fig. 15 present the results, showing relationships between flow against head, power, and specific gravity (SG). The head–flow curve (Fig. 13) for water at 50 Hz serves as a baseline, illustrating typical centrifugal pump characteristics: head decreases as flow increases. Slurry curves deviate from this baseline, requiring a higher head at equivalent flow rates due to increased density and resistance. Reducing the pump speed to 40 Hz significantly lowers both head and flow capacity, consistent with pump affinity laws.

The power–flow curve (Fig. 14) indicates that power demand rises linearly with flow under all conditions. Slurry pumping requires more power than water, with 20% Cw exhibiting the steepest slope and reflecting higher energy consumption. Conversely, operating at 40 Hz substantially reduces power requirements. The SG–flow relationship shows that water maintains a constant SG of ∼ 1.0, while slurry exhibits a slight SG reduction as flow increases. For 20% Cw, SG decreases from ∼ 1.5 to ∼ 1.25; for 10% Cw, it drops from ∼ 1.3 to ∼ 1.15. This trend suggests that mixing was improved and local concentration reduced at higher flows, which influences pump performance and power demand.

Overall, higher slurry concentration (20% vs. 10%) significantly increases power consumption and head requirements, while reducing pump speed (40 Hz) lowers hydraulic capacity and energy demand. These findings underscore the importance of accounting for slurry properties and operating conditions when designing and selecting pumps for slurry applications.

### 7.3 Calibration of flow meters

**Fig. 16:**
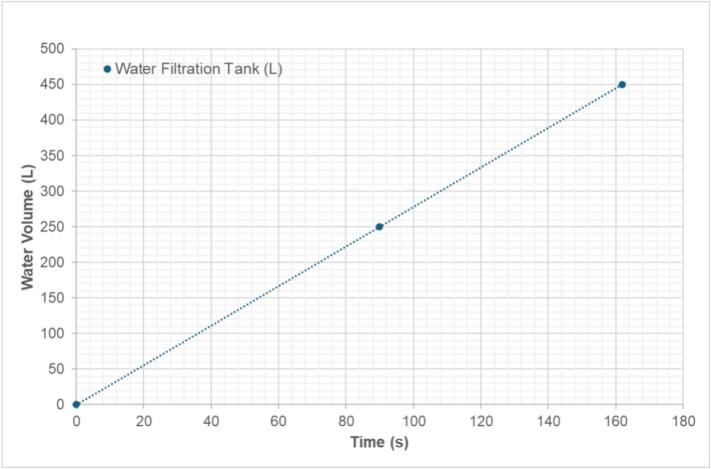
Flowrate calibration.

The calibration of the flow rate commenced by partially filling the hopper to approximately two-thirds of its nominal capacity (≈600 L). Subsequently, water was transferred to an empty filtration tank that serves as the collection vessel, with a total capacity of 450 L. The pump was operated at a set flow rate of 10 m^3^/hr, and the time required to fill the tank was recorded. The measured duration was 2 min 42 s, as illustrated in Fig. 16. Following this procedure, the pump and motor were engaged to initiate the circulation of water through the experimental test loop.

### 7.4 Calibration of vibration sensors

The calibration of the vibration sensors was conducted by first ensuring their secure installation on both the pump and motor, with particular attention to the dry and wet ends of the bearing assembly and the drive-end and non-drive-end of the motor. Before calibration, wireless communication between the sensors and the gateway was verified, along with sensor battery status. Upon initiating operation of the test rig, the sensors successfully transmitted vibration data to the gateway, which recorded the signals for subsequent analysis.

**Fig. 17:**
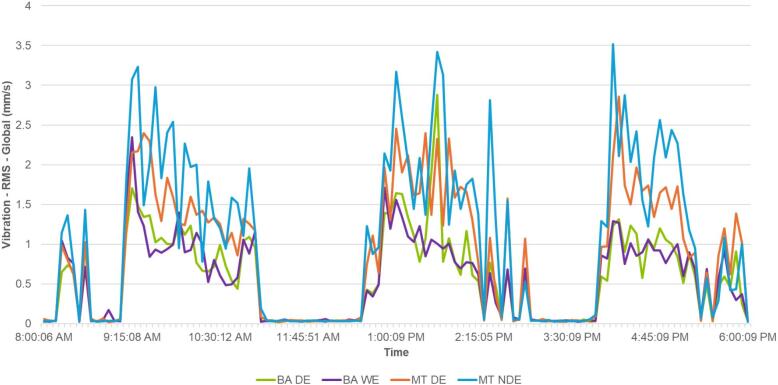
Axial vibration data across all sensors.

**Fig. 18:**
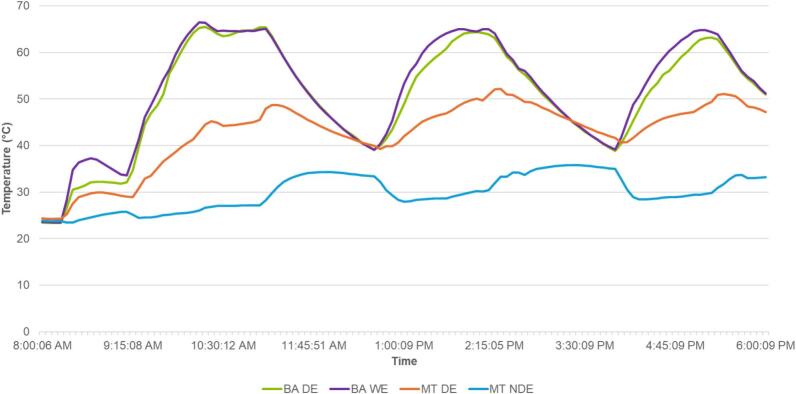
Temperature data across all vibration sensors.

**Fig. 19:**
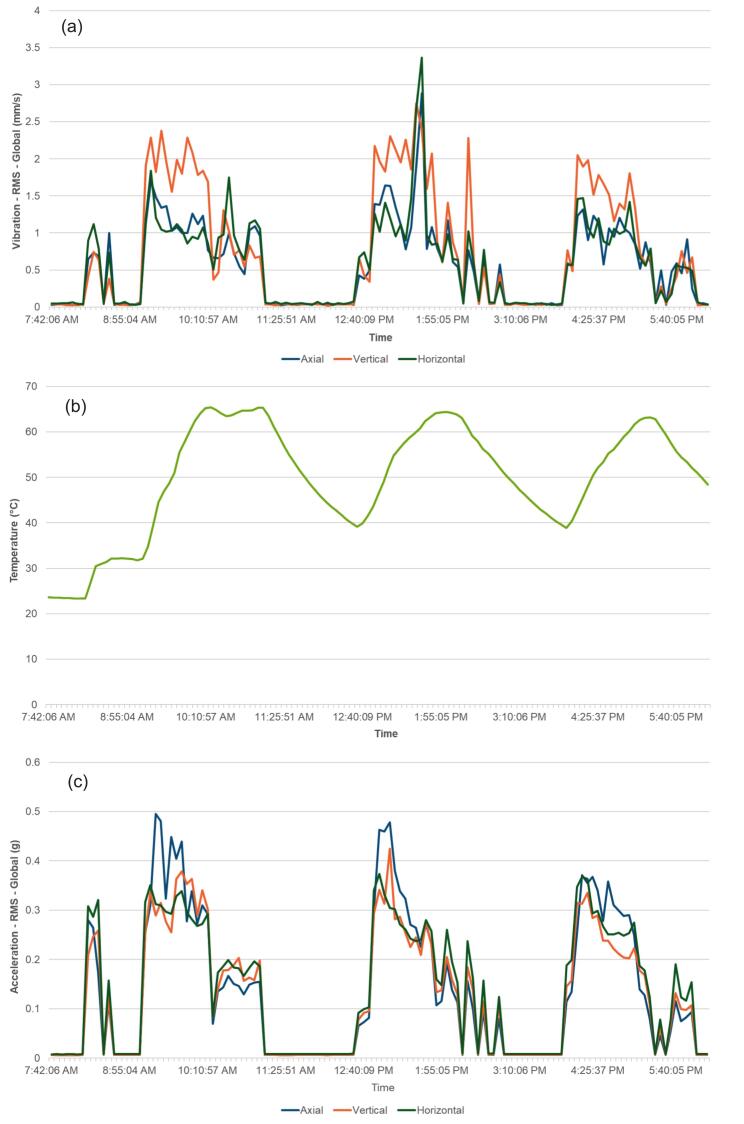
Vibration and temperature measurements for sensor BA‑DE showing (a) vibration (axial, vertical, and horizontal components), (b) temperature profile, and (c) acceleration (axial, vertical, and horizontal components).

Calibration consisted of verifying the stability of the signals over the network, the accuracy of frequency and temperature measurements, and the synchronization of data collection intervals. Following confirmation of connectivity, vibration and temperature data were uploaded to the cloud platform for real-time monitoring. This process ensured that the sensors provided accurate and continuous measurements for both pump and motor assemblies. Fig. 17 presents axial vibration data across all sensors, Fig. 18 illustrates surface temperature readings, and Fig. 19 provides a detailed view of sensor BA-DE, including axial, horizontal, and vertical vibration components alongside temperature profiles.

### 7.5 Wear sensor performance

The wear sensor (WearSense) installed on the test rig demonstrated stable, leak-free operation under varying conditions with both water and slurry media. Minor initial leakage, observed as small water drips, was effectively mitigated by applying sealant. Based on these observations, future installations should consider design enhancements, such as tapered configurations, to further improve sealing integrity and operational reliability.

The objective of this assessment is to evaluate mechanical fit, sealing integrity, and suitability for slurry service rather than to quantify pipe wear. Significant material wear is not expected during the testing period, and the wear measurement capability of the sensor falls outside the scope of the present work [29].

### 7.6 Water filtration unit performance

Following slurry testing, the water filtration unit was evaluated by diverting the slurry through the bypass line into the filtration system. The unit effectively separated solid and liquid phases, with sand particles retained within the geofabric bag (Fig. 9) while clarified water was discharged into the storage tank. The recovered water was subsequently recirculated to the hopper for reuse, and the collected sand was disposed of in accordance with safety protocols. This process demonstrated the unit’s capability to maintain efficient filtration and enable resource recovery for continued operational cycles.

### 7.7 Limitations and future work

The test rig is not intended for continuous long‑duration operation. Consequently, significant wear of pump components, piping, or instrumentation is not expected during the testing period reported in this study. Some sensors, such as vibration sensors, are battery powered and may require battery replacement over extended service life, with typical battery lifetimes on the order of several years.

The proposed slurry pump test rig is intentionally scoped to support pump‑level performance and condition assessment under slurry operation using digitally enabled measurement and analysis tools. As such, the rig is not intended to characterize slurry rheology, flow‑regime behavior, slurry transport scaling, or pipeline design phenomena. These aspects require specialized facilities and methodologies and fall outside the objectives of the present work.

The study does not aim to extrapolate hydraulic performance to full‑scale slurry pumping systems using similarity laws or dimensionless scaling relationships. Instead, scalability is addressed through the selection and application of digital monitoring and analysis methodologies that are transferable across pump sizes and operating contexts. Techniques such as vibration‑based condition monitoring, performance trending based on measured operational data, and sustainability‑conscious indicators can be applied across slurry pumps and are largely independent of pump size. Accordingly, the focus of this work is on methodology development and validation rather than application‑specific hydraulic characterization.

Future work will extend the use of the test rig to a broader range of slurry pumping conditions and operating scenarios. In particular, planned studies will incorporate worn pump components to evaluate wear‑induced performance degradation using digital monitoring techniques. This will enable systematic investigation of how performance, efficiency, and condition indicators evolve with component wear, supporting sustainability‑driven optimization of pump operation and maintenance strategies through improved timing of interventions and better utilization of asset life [14].

## CRediT authorship contribution statement

**Alan Varghese:** Writing – original draft, Visualization, Validation, Project administration, Methodology, Investigation, Formal analysis, Data curation, Conceptualization. **Paul Huggett:** Writing – review & editing, Validation, Methodology. **Evert Lessing:** Writing – review & editing, Supervision, Resources, Funding acquisition, Conceptualization. **Ghulam Mubashar Hassan:** Writing – review & editing, Supervision. **Ali Karrech:** Writing – review & editing, Supervision, Resources, Funding acquisition, Conceptualization.

## Declaration of competing interest

The authors declare that they have no known competing financial interests or personal relationships that could have appeared to influence the work reported in this paper.
